# Family quality of life and children with disability in Ethiopia: The role of support providers

**DOI:** 10.4102/ajod.v12i0.1124

**Published:** 2023-02-16

**Authors:** Julia Jansen-van Vuuren, Solomon Dawud, Rosemary Lysaght, Beata Batorowicz, Heather M. Aldersey

**Affiliations:** 1 School of Rehabilitation Therapy, Faculty of Health Sciences, Queen’s University, Kingston, Canada; 2 Community Based Rehabilitation, University of Gondar, Gondar, Ethiopia

**Keywords:** family quality of life, families, children with disabilities, support providers, Ethiopia, support, spirituality

## Abstract

**Background:**

Family quality of life (FQOL) is an important outcome for families of children with disabilities globally and provision of support is associated with enhanced FQOL. However, FQOL research primarily focuses on conceptualisation and measurement, and originates from high-income contexts despite the fact that most children with disabilities live in low-income countries.

**Objectives:**

The authors examined how Ethiopian disability support providers practically contribute to meeting the needs of families of children with disabilities to enhance FQOL.

**Method:**

Building on a previous study exploring Ethiopian families’ perspectives on FQOL, the authors used an exploratory descriptive qualitative approach to interview various support providers. Interviews were conducted virtually (because of the coronavirus disease 2019 [COVID-19] pandemic) in English or with interpreting assistance. Audio-recorded interviews were transcribed verbatim and analysed thematically.

**Results:**

Support providers affirmed what families had described as important for FQOL – spirituality, relationships, self-sufficiency – and recognised their enormous support needs. They described various ways to support families – emotionally, physically, materially and informationally. They also expressed challenges and their need for support to meet families’ needs.

**Conclusion:**

Ethiopian families of children with disabilities need holistic support that incorporates spirituality, the whole family’s needs and disability awareness-raising. Collaborative and committed engagement from all stakeholders is necessary to support Ethiopian families to flourish.

**Contribution:**

This study contributes to global understandings of FQOL and describes practical approaches to support families of children with disabilities in an African context. The findings of this study highlight the influence of spirituality, relationships, self-sufficiency, poverty and stigma and the need for holistic support and disability awareness-raising to enhance FQOL.

## Introduction

Family quality of life (FQOL) as a construct of interest with regard to disability has developed in recognition of the effect of disability on the collective family unit (Brown, Kyrkou & Samuel 2016; Samuel et al. 2012). Zuna et al. (2010) defined FQOL as ‘a dynamic sense of well-being of the family, collectively and subjectively defined and informed by its members, in which individual and family-level needs interact’ (p. 262). Building on the theory and framework of Zuna and colleagues, Chiu et al. (2013) updated the FQOL framework to emphasise family strengths as well as needs. According to this FQOL theory, individual-member and family-unit factors interact with each other and support factors (at individual and family levels) within the context of systems, policies, programmes and societal values, to determine FQOL and other child outcomes.

Research on FQOL has primarily occurred in high-income contexts, even though globally, 80% of children with disabilities live in low-income contexts (United Nations International Children’s Emergency Fund [UNICEF] 2020). Contextual values, norms and environments play a critical role in FQOL; therefore, understanding FQOL from specific cultures and contexts is crucial to developing a global understanding of FQOL and providing appropriate support. In addition, FQOL research originated with a focus on families of children with intellectual and developmental disabilities (IDD) (Brown et al. 2016), and this continues to comprise the majority of FQOL literature. Although these findings provide important insights into FQOL, further research is needed with families of children with diverse abilities and backgrounds.

### Family quality of life and support

Despite variable definitions of support in research literature, this study refers to support broadly as strategies and resources provided to families of children with disabilities to contribute to meeting their needs (Aldersey 2012; Chiu et al. 2013; Kyzar et al. 2012). Global research evidence highlights the positive association between FQOL and support (Boehm & Carter 2019; Kyzar et al. 2012; Mora, Ibáñez & Balcells-Balcells 2020; Zuna, Brown & Brown 2014); however, researchers and practitioners need to better understand how the type and extent of support provision contributes to FQOL. Support from formal service providers that is strengths-based and family-centred with positive, respectful and trusting partnerships between families and professionals is crucial for promoting FQOL (Balcells-Balcells et al. 2019; Summers et al. 2007; Vanderkerken et al. 2019). Furthermore, informal support from family, friends and community is significant for enhancing FQOL, and can be even more crucial for families in low-income contexts with limited professional providers (Nuri, Batorowicz & Aldersey 2019). A recent scoping review on factors contributing to FQOL in African contexts highlighted financial support, respite, transport and educational support for children with disabilities as well as employment support for parents as important for enhancing FQOL (Jansen-van Vuuren et al. 2022). Informational support around prognosis and care for their children, provision of rehabilitation and medical services, as well as emotional support from family and friends or professional counselling were also found to contribute to better FQOL. However, FQOL studies in African contexts confirm the overall inadequacy of both formal and informal support for families of children with disabilities (Ajuwon & Brown 2012; Aldersey et al. 2017; Jansen-van Vuuren et al. 2021). Further research is needed to understand how the provision of support can positively affect families of children with disabilities, especially in low-income settings.

### The current study

Whilst international FQOL literature is growing, research remains primarily focused on conceptualisation and measurement; implementation of research findings, particularly around how to practically support families, is necessary (Edwards et al. 2018; Zuna et al. 2014). This is especially true in low-income countries where families often face overwhelming challenges with few social safety nets; they urgently need culturally relevant and accessible support. Therefore, this study aimed to explore how local disability support providers can practically contribute to meeting the needs of families of children with disabilities (hereafter referred to as ‘families’) in Ethiopia, thus enhancing FQOL. Prioritising Ethiopian families’ conceptualisation of FQOL rather than using FQOL frameworks from vastly different contexts (i.e. high-income countries), this study builds on a previous study from families’ perspectives, described in more detail in an earlier publication. (Jansen-van Vuuren et al. 2021). Based on this understanding of FQOL, the authors sought to answer the research question – how can Ethiopian disability support providers address needs identified by families and contribute positively to FQOL?

## Research methods and design

### Study design

The authors used an exploratory descriptive qualitative approach, appropriate for research in areas with limited previous research (Hunter, McCallum & Howes 2019).

### Research team

All the researchers were affiliated with universities in departments involving the fields of rehabilitation and community-based rehabilitation (CBR). Although only one author (S.D.) was Ethiopian, the research team all had prior experience working or researching in an Ethiopian context. The first author (J.J.-v.V), at the time of the study a PhD candidate, spent time in Ethiopia in the region where data were collected, and received invaluable and ongoing input and guidance from Ethiopian colleagues. With his insider knowledge of the local context, culture and language, as well as specific experience in CBR working with support providers and families of children with disabilities, S.D. coordinated recruitment and provided important insights throughout the research process including data collection, analysis and interpretation of findings. J.J.-v.V directed the research, conducted interviews and did initial data analysis and drafting of the manuscript, all of the authors contributed to the analysis and interpretation of findings and writing of the final manuscript.

### Setting

**FIGURE 1:**
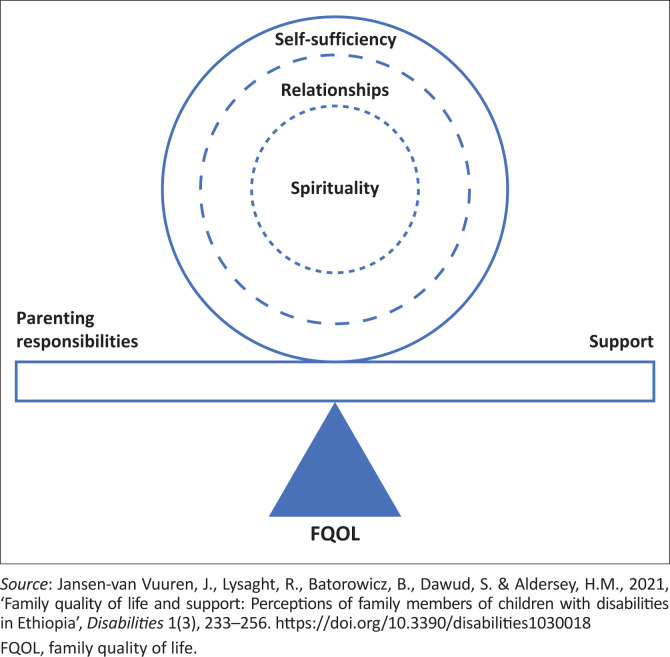
Visual representation of family quality of life as described by Ethiopian families of children with disabilities.

This study was based in Gondar, northern Ethiopia, following an earlier study with family members of children with disabilities in the same region. To ground their studies in an African context and to inform their interview guides, the researchers first conducted a scoping review to identify factors contributing to FQOL in African contexts (Jansen-van Vuuren et al. 2022). Subsequently, the authors explored the perspectives of Ethiopian families to understand how they conceptualise FQOL and their support needs (Jansen-van Vuuren et al. 2021). Both studies highlighted spirituality as foundational to FQOL. This is perhaps unsurprising with regard to Ethiopia specifically as it is a deeply religious country with most of the population identifying as orthodox Christian, followed by Muslim, then protestant Christian (CIA 2022). The families also described both loving and supportive relationships as well as stigma and exclusion; stigma was another prominent finding in the scoping review. Many families valued self-sufficiency (primarily through education and employment) as important for FQOL, as well as having enough to help others. Amidst their parenting responsibilities, poverty was a major challenge, and although families acknowledged the counteracting benefits of support, their overwhelming need for more support was undeniable. Figure 1 provides a visual representation of these findings.

### Study population and sampling strategy

This study included participants who provided support in some capacity to children with disabilities (age 0–18) or their families, regardless of disability type. Ethiopia has signed the United Nation’s Convention on the Rights of Persons with Disabilities (UNCRPD) and therefore, has legally adopted their broad definition of disability, including children with physical, mental, intellectual and sensory impairments which, along with environmental barriers, hinder their full participation (UN 2006). The authors purposefully sampled for a broad range of participants with diverse roles with families, various educational backgrounds, and both male and female. Hence, they used the term ‘disability support provider’ (hereafter, ‘provider’ or ‘participant’), to encompass the broad range of support provision, beyond specific services.

### Data collection

Author S.D., who coordinates the University of Gondar (UoG) CBR programme, directed participant recruitment as he is familiar with local organisations and individuals who work with families. Participants initially received a consent letter and information document to reflect on, outlining findings of our previous research of families’ perceptions of FQOL (Jansen-van Vuuren et al. 2021). Participants were invited to an interview through email or personal communication from the CBR office and the authors confirmed verbal consent prior to interviewing. Because of coronavirus disease 2019 (COVID-19), all interviews were conducted using Zoom and lasted between 60 min and 90 min. The authors developed a semi-structured interview guide based on their previous studies. Participants who chose to be interviewed at the CBR centre (more reliable internet access) received a modest remuneration for transport (300.00 Ethiopian Birr, approximately 5.50 USD). Twelve participants chose to interview with an Amharic interpreter, a skilled bilingual UoG faculty member from the English Department. All interviews were audio-recorded and transcribed verbatim. The authors were guided by ‘information power’ to determine data sufficiency rather than saturation (Braun & Clarke 2021). Information power considers the study’s aim, sample specificity, established theory, quality of dialogue and analysis strategy, recognising that exploratory studies do not aim for a complete description but rather varied accounts of a new phenomenon (Malterud, Siersma & Guassora 2015).

### Data analysis

The researchers based their data analysis on Braun and Clarke’s approach to reflexive, qualitative thematic analysis (Braun & Clarke 2006; Braun et al. 2019), including familiarising themselves with the data, generating codes, constructing and revising themes, defining and naming themes, and finally writing the report as presented in this manuscript. However, after commencing inductive coding initially, they diverged slightly by sorting codes deductively into themes related to aspects of FQOL derived from Ethiopian families’ perspectives. The researchers recognised that using a theoretical framework deductively does not reflect a pure reflexive thematic analysis (Braun & Clarke 2022); however, this approach aligned with the research purpose to explore how providers can practically contribute to supporting FQOL as described by families. The researchers also observed other inductive themes outside of this FQOL framework. All included quotes are in English, either participants’ direct words or the interpreter’s translation of Amharic responses (with light grammatical editing).

### Trustworthiness

To improve rigour the first author maintained a reflexive journal and audit trail, documenting the research process and reflecting on potential sources of bias, especially as a foreigner to the local context (Annink 2017; Resch & Enzenhofer 2018). The authors were guided by Ethiopian colleagues’ recommendations, particularly author S.D., in relation to data collection and analysis to ensure cultural sensitivity and feasibility. Throughout the interviews the interviewer clarified comments that were unclear; however, they did not conduct member checks with participants after analysis, because of time and budget constraints and recognising that member checking is not always appropriate or necessary for all qualitative research (Motulsky 2021). During data analysis, the authors used triangulation, where two authors independently coded several transcripts before discussing the findings to promote ideas and incorporate different perspectives. All authors discussed and provided feedback on the findings and written report.

### Ethical considerations

Queen’s University Health Sciences and Affiliated Teaching Hospitals Research Ethics Board and the Institutional Ethical Review Board of the University of Gondar provided ethical clearance prior to commencing the study: #6028643.

## Findings

A total of 18 participants were interviewed. Five were affiliated with a university in rehabilitation, social work and special needs and inclusive education. Four were affiliated with CBR as supervisors or coordinators (with prior fieldwork experience), three worked for government departments in Labour and Social Affairs and Women and Children’s Affairs offices, and two were government-employed special-needs education coordinators. Another three participants worked with Organisations of Persons with Disabilities (OPDs), and one with a non-profit charity. Table 1 provides additional demographic details.

**TABLE 1:** Demographic characteristics of study participants.

Characteristics	*n* = 18	%
**Age of participant (years)**		
25–29	3	16.7
30–34	3	16.7
35–39	5	27.8
40–44	4	22.2
45–49	1	5.6
50+	2	11.1
**Sex of participant**		
Female	9	50.0
Male	9	50.0
**Highest level of education**		
Grade 12	1	5.6
Certificate	1	5.6
Diploma	1	5.6
Bachelor’s degree	8	44.4
Master’s degree	7	38.9
**Affiliation**		
Rehabilitation professional	3	16.7
Social work	1	5.6
CBR	4	22.2
Special needs and/or Inclusive education	3	16.7
Government	3	16.7
OPD	3	16.7
Charity	1	5.6
**Lived experience of disability**		
Yes	3	16.7
No	15	83.3
**Interpreter present during interview**		
Yes	12	66.7
No	6	33.3

CBR, Community-Based Rehabilitation; OPD, Organisation for Persons with Disabilities.

Most participants affirmed what families in our previous study had described as important aspects of FQOL – spirituality, relationships (within the family and community), self-sufficiency – as well as the need for support to counter the many responsibilities and challenges that families face. Beyond this FQOL framework, participants shared their own challenges, frustrations and disappointments as they support families in an Ethiopian context.

### Incorporating and supporting spirituality

All but one participant affirmed the centrality and significance of spiritual beliefs in Ethiopian families’ lives. Many highlighted how spirituality positively influences FQOL e.g.:

‘I strongly believe that when families are faithful, when they have strong spiritual beliefs, they tend to support the children … this supports the family and helps the family to cope with the challenges.’ (014, female, OPD)

Providers recognised the importance of respecting and building on spiritual beliefs and values to work with families and provide holistic support. One rehabilitation professional described how he counsels and encourages families by sharing how others cope through their spiritual beliefs. Others spoke about drawing on families’ spiritual beliefs to promote acceptance and commitment to their children with disabilities: ‘What we do is to tell the mother to stay strong and keep on supporting the child so that God also supports the family’ (007, female, CBR).

Many participants believed that spirituality – specifically religious teaching – promotes inclusion, equality and value of children with disabilities, and should be encouraged. Therefore, participants described religious leaders’ important influencing role:

‘… the teaching of the religious fathers has a positive influence, to see the children in a way we see children with no disabilities … there is a positive connection between the way the families treat the children and the teachings from the church and the mosque.’ (017, male, charity)

A number of participants also described how families take their children to holy water in search of healing, seeing this as a positive aspect of spirituality.

All participants acknowledged the negative effect of stigma and discrimination on FQOL, often because of beliefs about spiritual causation of disability (e.g. God’s curse or punishment). Participants described these negative beliefs as being predominantly cultural and because of limited disability-awareness, as opposed to being religiously founded:

‘In mosques or church … they teach there is no problem, please support [*children with disabilities*] … So there is no direct connection with a curse or sin … I think it’s culture, the problem that comes … they mix the cultural attitudes with the spiritual.’ (008, male, CBR)

To counter these stigmatising attitudes, participants identified education and awareness-raising as one of their primary roles in supporting families: ‘… [we] give awareness to the parents about the cause of disability and it is not a spiritual problem’ (003, female, rehabilitation professional). Religious institutions were sometimes a platform for such awareness-raising, for example, CBR workers taught about disability during church programmes. Providers also described how, through building rapport and trust with families and gently guiding them, they can counteract negative disability beliefs.

### Supporting relationships

#### Family relationships

Participants acknowledged family and social relationships within Ethiopian culture as particularly crucial for families:

‘… in Ethiopia, the social interactions are different from other countries, for example from Europe or in Western countries. Family interactions are more highly valued in Ethiopia … the life of one person depends on the other here.’ (002, male, rehabilitation professional)

Another participant stressed that stable, loving and committed family relationships are necessary for FQOL:

‘The child who grows up with his mother and his father has an opportunity to develop positive social and psychological wellbeing in the family. And also there is a responsibility shared with the father and the mother in the family. So it has a positive contribution for the quality of life for the parents and also for the children with disability.’ (004, male, social worker)

To nurture positive family relationships, participants described how education and awareness-raising are important, so families accept and appropriately support their children with disabilities. Reassurance of support was one way to encourage acceptance. One therapist explained how acceptance was a prerequisite for families to properly engage in treatment:

‘… those who accepted [*the disability*] are usually less stressed, and they don’t wander here and there … those who do not accept their children’s disability or those who are looking for a quick fix or complete recovery, they may not accept what I’m telling them, or they may not accept my therapy.’ (001, male, rehabilitation professional)

Other participants explained the importance of educating families around communication approaches (e.g. sign language) to improve relationships.

Providers described many families as committed and loving towards their children. One CBR supervisor shared:

‘We sometimes challenge the mothers intentionally, whether they actually love their children or not … almost all of the mothers love their children. So they try their best to bring the children up on their own.’ (007, female, CBR)

However, participants observed that many families also had low expectations of their children with disabilities, leading to poorer family relationships:

‘Almost all mothers expect their children to grow up and support them … when they have to deal with disabilities, then they see no benefits in bringing them up because they fail to provide the support that the family expects … And the mothers also become desperate that these children would not be there for them as they grow old, so that’s a cause of discrimination.’ (015, female, government officer)

Participants described their role in advocating for the value and contribution of children with disabilities; one way was by highlighting successful role models. Another participant explained the need to educate families about promoting their child’s function and independence by encouraging participation in household activities rather than being overly protective.

Many participants acknowledged that disability strained family relationships, often resulting in marital conflict or the father abandoning the family. Providers described various approaches to restoring broken family relationships through counselling and mediation:

‘… our [*CBR*] fieldworkers, they know the families around the *kebeles* [smallest administrative units]. So we use them to negotiate, to deal between husband and wife … In addition to that we use priests and other elderly people that live around them, just to counsel both wife and husband.’ (008, male, CBR)

In extreme cases, some participants were involved in court cases to ensure that fathers supported their children.

Holistic support was identified as important for strengthening family relationships and promoting FQOL:

‘[*Previously*] I was so focused on the medical part. I was not focused on their feelings or parents’ struggles … But now I’m more holistic and I even sometimes talk with parents, and I ask them what are their challenges and what they think about this …’ (001, male, rehabilitation professional)

Several participants also mentioned the importance of sibling relationships and how siblings can promote independence and the value and contribution of children with disabilities:

‘Earlier the siblings used to pity the children with disabilities so that they didn’t allow them to do anything at home … But recently, siblings are supporting the children so that they become independent.’ (014, female, OPD)

However, providers did not elaborate on how they can support siblings or other family members specifically.

#### Community relationships

Community relationships were another key component of FQOL for Ethiopian families, and all participants confirmed this significance. Participants described mixed relationships between communities and families:

‘Most of the societies we work with are cooperative, they even help us in identifying these children with disabilities. But there are cases where the members of society discriminate against these families.’ (006, female, CBR)

In some cases, community members seemed to have good awareness about disability (better than some disability workers). However, stigma and negative attitudes from the community were unanimously identified as a major barrier to FQOL. Participants acknowledged that changing negative societal attitudes is crucial for full participation:

‘… all children with disabilities need to be involved in every social participation in the community without any discrimination and isolation. But the reality is not that … The source of every change is attitude. If the attitude of the parents is changed and if the community attitude towards children with disabilities became positive … it would allow them to participate in every aspect in the community without any discrimination and isolation based on their impairment or disability.’ (004 female, social worker)

Participants described various ways they raise awareness and educate the community, as well as families, to improve relationships and acceptance of children with disabilities. However, they acknowledged, ‘… it’s an ongoing process. It takes time and we are working on that, and we are seeing improvements’ (015, female, government officer). Specifically, providers discussed how they reach people using public platforms, including coffee ceremonies, marketplaces, hospitals, churches, and through community leaders and radio:

‘We use *woreda* [district consisting of several *kebeles*] and *kebele* meetings. And we also reach the community by using platforms created by the churches. And we also have focal persons.’ (016, male, government officer)

Several participants observed that education and literacy positively influence community attitudes towards disability. One approach to raise disability-awareness was by highlighting everyone’s vulnerability and promoting disability prevention:

‘… it’s important to work on attitudinal change because disability is imminent. We all are vulnerable. So at some time, at some point we might all become a person with disability.’ (012)

Providers also attempted to highlight the collective benefit of including children with disabilities for the country’s overall development:

‘… the community should change this [*negative*] perspective in order to allow those children with disabilities to be part of the community and work and contribute their skills and knowledge for the good of the community.’ (004, male, social worker)

One participant mentioned that research is also important to supporting advocacy and changing negative attitudes as well as raising community awareness.

Participants described how they support families’ community integration. For example, several participants described the importance of teaching children with disabilities functional skills and basic hygiene to improve acceptance into society. One participant shared how they support families to protect children from harm in the community:

‘… the families try to protect and care for the children with disabilities, especially children who are older than 14 years … there was a child who was raped and impregnated by a school guard. So we teach the families to protect children with mental impairments, they work with us in protecting their children.’ (014, female, OPD)

In addition, providers explained how they promote peer-support particularly amongst mothers, which provides both practical (economic) and psychological and emotional support:

‘… we form groups of mothers having the same situation of having children with disabilities. We create a platform for them so that they discuss and share information about their children. Sometimes mothers hide their children, thinking that their children have the worst case, but after they discuss amongst themselves, they happen to discover that there are even worse cases, worse than theirs.’ (006, female, CBR)

### Supporting self-sufficiency

Nearly all participants agreed that most families are hardworking and committed; they strive to be self-sufficient, and they value education and employment to achieve this, but they require support. One participant acknowledged the risk of cultivating dependency in providing support, but found that this is uncommon.

#### Education of children with disabilities

Although the situation is improving, many participants recognised that children with disabilities need more support for school participation, benefiting the child as well as the whole family. Stigmatising attitudes at home and school remained a barrier for school participation:

‘First of all there is a problem. Starting from the mothers, they don’t have knowledge whether the kids can go to school and be taught or not. And the second [*problem*] is they don’t believe the kids can be taught and get knowledge and will be normal … the third [*problem*] is that there aren’t any schools which include them.’ (003, female, rehabilitation professional)

Particularly in rural areas, many families are farmers, prioritising physical labour necessary for survival, over their children’s education and academic learning. In addition, parents often do not have the time, capacity or resources to take their children to school or support them at school when they need to work long hours in the fields or in other low-paying jobs (e.g. baking *injera* [traditional flat bread], washing and cleaning for wealthier families). Providers described how, through dedicated awareness-raising and advocacy, more children with disabilities are now attending schools:

‘Before we started the programme, there were many children who were deprived of the right to go to school because the families and the neighbours used to believe that the deaf, the blind, and children with any sort of disability cannot learn. We have worked closely with the families and the society, and we have also tried to put these children in school ourselves by consulting with the families.’ (006, female, CBR)

Another awareness-raising approach was celebrating special days such as Children with Disabilities Day. Again, participants shared how highlighting successful role models encourages families to educate their children:

‘Some of the families have witnessed that some children with disabilities have succeeded in their education and they have also been recruited and are doing well in their jobs … some families are motivated by these successful people. So they put their children in school to become as successful as their role models.’ (009, male, OPD)

Participants shared approaches to support education, including providing basic financial support, teaching children communication and functional skills, and promoting independence. They also spoke about the need for assistive devices and material support:

‘… there should also be access to schools … for example, by giving blind students white canes so that they can go to school. And assistive devices in general should be second of the priorities. And the next should be stationery, because these students might join schools, but still they need that kind of support.’ (013, female, government officer)

Participants also highlighted that inaccessible schools need environmental modifications:

‘We have tried a lot to create a conducive environment, to create access within the school compounds so that they can use wheelchairs and crutches. And we’ve also worked with the schools so that they can fix bathrooms so that they are accessible for children with disabilities.’ (005, female, CBR)

One CBR supervisor described her efforts to overcome numerous barriers, including negative attitudes, inaccessible environments and financial barriers, to successfully support a child with a physical disability pursue his education.

Participants described various approaches directed at schools and governments to promote education of children with disabilities. One participant explained that teachers need more training and expertise to support children with complex needs. Another participant recognised that teachers are overburdened and need better support and incentives. Several participants hoped for more schools especially in rural areas. A special-needs education coordinator shared how she supports close relationships and collaboration between families and schools through regular communication:

‘… we evaluate changes every month with the families … we are always in communication. The families also ask us about ways to support their children … there is a strong bond between the families, the children, and the school.’ (011, female, special needs educator)

Participants described both special-needs schools and inclusive education, although the differences between them were sometimes unclear. One educational provider explained how inclusion has not yet been fully realised:

‘Inclusion is of course the new idea here in Ethiopia. Still, there is an argument that we are not on track for inclusion sometimes, just a kind of integration rather.’ (018, male, special needs educator)

Children with physical disabilities were generally directed into mainstream schools, whilst children with other disabilities (e.g. sensory, communication or intellectual impairments) were initially placed in special education settings and then expected to transition to mainstream classes, often with minimal support.

#### Parental employment and work

Many participants discussed the importance of employment and income-generation for families and some of the challenges in obtaining and maintaining work, particularly for mothers. The constant care needs of children with disabilities were a major barrier for mothers and several participants mentioned how quality childcare could alleviate this; however, they acknowledged the limited availability, affordability or even welcome for children with disabilities. Mothers often faced stigma when attempting to work, requiring providers to advocate and raise awareness:

‘Most of the mothers are from low-income families, so they are supposed to do laundry for people, or they sometimes bake *injera* [*traditional flat bread*] for other people. So people have concerns in terms of hygiene … they will not be willing to have them work for them … what we do is to consult people to teach other people that … [*disability*] is not communicable.’ (007, female, CBR)

Participants shared various other ways they can support employment, including vocational skills-training and linking to job opportunities. Furthermore, providing start-up capital was an important support for families to initiate their own business or income-generating endeavour:

‘Once we identify [*poor families*], we make sure that these families get start-up capital. And by doing this they can provide for their family, and they can provide all the basic needs for the children with disabilities.’ (013, female, government officer)

One participant described a collaboration with local businesswomen to provide loans to mothers so that they can work and not beg. Establishing peer-support groups also helped mothers to participate in income-generating activities. How to manage their income was another area for support. However, one participant highlighted the need to consider each family’s situation and provide choice in decision making:

‘… before we provide [*the mothers*] with money or finance, we consult with them to make the right choices so that they can do the appropriate work while they feed and care for their children. So it all depends on the mothers and it’s for them to decide the kind of work they would like to do.’ (005, female, CBR)

### Empowering families to manage their responsibilities

Apart from education and employment, participants described several other approaches to support families amidst their many challenges and responsibilities. Firstly, at the child-level, some participants shared how they promote function and independence through specific interventions (e.g. physiotherapy exercises) and training. Connected with this, some participants taught parents treatment techniques and how to prevent complications (e.g. contractures, spasticity and atrophy). However, one participant highlighted the need to carefully clarify possible child-related outcomes to minimise unrealistic expectations and disillusionment with interventions. Providing appropriate assistive devices was another way to improve children’s participation and FQOL:

‘… it’s not easy to get a wheelchair, it’s not affordable, so offering a wheelchair for the child will enable them to connect and establish a connection between himself and the society and family.’ (015, female, government officer)

Participants also described how they link families to other supports if they cannot provide them. Early identification, screening and referral of children with disabilities were important, mainly through the hard work of community fieldworkers and promoting community disability-awareness.

Secondly, at the family-level, participants affirmed the need to involve families in interventions. They recognised the importance of supporting families more broadly, not just the children. Providers particularly focused on supporting mothers as they bear the brunt of responsibility and sometimes require basic support just to survive:

‘Always [*the mother*] carries the child with her. Because of that, she needs support. It can be food, it can be shelter or it can be any clothing, because she doesn’t have any work to earn money.’ (003, female, rehabilitation professional)

Housing was another needed support, as families were often discriminated against or unable to afford rent:

‘Most of the families don’t have their own shelter, even if they want to rent the house there are two problems still. One is they can’t afford to rent a house, and the second case is that the people who let them in, they don’t want to have a family who has a child with disability. Even if they do manage to rent a house, once the owners find out, they automatically tell them to evacuate, to move out.’ (007, female, CBR)

Most participants described families’ poverty and enormous needs, sometimes even forcing them to give up their children for adoption:

‘… the families are fond of the children. They are compelled to submit the children to the [*charity*] on the basis that they cannot afford to raise their children on their own because they have economic problems.’ (017, male, charity)

Participants recognised their role in providing financial and material support to families including food, clothing, educational materials or start-up capital for a business.

Thirdly, participants described their role in advocating for children with disabilities and their families at a higher governance-level. Several participants highlighted policy gaps: ‘… the primary responsibility of the office I’m working in is creating awareness, because some policies and legislations are not implemented’ (016, male, government officer). Another participant highlighted the need for providers to empower OPDs so they can advocate and raise disability awareness themselves. The urban–rural divide was another highlighted issue, where some providers called for decentralisation of services and more support for rural families. Additionally, transportation in rural areas was a barrier for families and service providers: ‘… we are unable to reach the children with disabilities and their families because of lack of transportation’ (014, female, OPD). Several providers dreamed about having focused, specialised support for children with disabilities and their families, for example a comprehensive rehabilitation centre. Participants considered collaboration amongst multiple stakeholders as crucial to address families’ multidimensional needs:

‘It’s essential to collaborate; that’s the families, the society and the government working closely … we need to create awareness so that stakeholders support the children with disabilities to become self-sufficient and independent … it’s unthinkable to secure development [*for Ethiopia*] without the inclusion of the 20 million people with disabilities.’ (010, male, OPD)

One participant highlighted why everyone should take responsibility:

‘… [*disability*] is the issue of all organisations because disability can happen even within a fraction of seconds, can’t it? … We don’t have any guarantee to live without disability. So the issue is for all human beings.’ (018, male, special needs educator)

### Providers’ challenges

Participants of varying expertise were motivated and committed to supporting families to enhance FQOL. However, many highlighted overwhelming needs and some expressed disappointment and frustration in being unable to meet such huge demands:

‘I talked with CBR workers and most of them are disappointed, and some of them are frustrated and yeah, me too. It’s very difficult because you go there and there is a need and you can’t do anything.’ (001, male, rehabilitation professional)

A lack of trained professionals, limited budgets and negative attitudes were further sources of frustration. Another participant explained that lack of prioritisation and ineffective disability policies were the reason for limited budgets:

‘The government has a priority in providing a budget. So if they don’t consider the issues of children with disability or, in general, persons with disabilities get minimal attention, there is no possibility to get a huge amount of budget that could provide the full services for the empowerment of children with disabilities and the parents with disabilities.’ (004, male, social worker)

Limited budgets and personnel hindered efforts for early identification, screening and intervention:

‘I also regret contacting children who contact us late because some of them could have been treated if they had approached us early … there are some occasions which are sad.’ (013, female, government officer)

Other sources of frustration were equipment shortages, inadequate maintenance and inappropriate assistive devices. Several participants shared how they faced discrimination because they worked with children with disabilities:

‘… things have changed a lot now, but when we started … we did not even feel comfortable doing the job because people used to tell us that it’s not worth doing it … that we should give up the job and stop fooling around with fools.’ (007, female, CBR)

Another participant expressed frustration with the education system that restricted highly qualified teachers from using their skills because their work in special education was undervalued. Even when one participant provided training to support teachers working with children with disabilities, she was disappointed that they expected payment. Several providers from different backgrounds expressed a need for more training and professional education to enhance support provision. One participant specifically indicated they need training to write funding grants to implement better projects.

Although participants recognised that multiple stakeholders must take responsibility for supporting children with disabilities and their families, some felt government was not doing enough:

‘The government is highly responsible for [*providing support*]. The government should set a platform or systematic arrangement … The policies, the programmes, the guidelines should clearly state the needs of children with disabilities and the required resources and the amount of budget that is required to fulfil those resources for children … the government should take the lion share.’ (004, male, social worker)

Another participant argued that the overarching need is attitude-change, but it is a time-consuming, often frustrating endeavour universally:

‘… you ask what is the challenge for inclusion; people can mention many challenges, like lack of resources, you know, but the umbrella issue is attitude. If we change the attitude, it is a person who allocates budget, isn’t it? It is a person who provides resources … attitudes cannot be changed overnight. It takes time, takes time not only in our country, even in your country, in developed countries also, the problem of the issue of attitudes is not addressed.’ (018, male, special needs educator)

## Discussion

Recognising the imperative for practical action to address contextual needs identified by families themselves, we spoke to various local support providers involved with Ethiopian families of children with disabilities to explore how they can positively contribute to FQOL. Participants affirmed that spirituality, relationships within the family and community, and self-sufficiency (primarily through education and employment) are important for Ethiopian families’ FQOL. They also reiterated the enormous need for support amidst multidimensional challenges that families face. Participants described various ways that they support families materially, emotionally, physically and through providing information (Kyzar et al. 2012). Beyond the FQOL conceptual framework derived from Ethiopian families’ perspectives (Figure 1), participants shared struggles, frustrations and disappointments in their attempts to support families, and their own need for support and collaborative engagement from stakeholders, particularly the government. Participants’ level of experience and expertise (Table 1) appeared to shape their insightful perspectives adding credibility to the findings.

### Spirituality and family quality of life

Although research indicates that spirituality is a crucial domain for well-being and a major contributor to FQOL (Boehm & Carter 2019; Poston & Turnbull 2004), formal support providers can overlook or avoid this dimension (Gaventa 2012), especially in western contexts where religion and spirituality can be perceived as purely private. Boehm (2022) argues that a theological reflection on FQOL is necessary (but overlooked) because relationship with God gives all humans value and meaning and is important for flourishing. This study confirms other FQOL research in African contexts where spiritual beliefs are pervasive and critical for families’ well-being and a major source of support for coping with the challenges of raising children with disabilities (Ajuwon & Brown 2012; Aldersey et al. 2020; Jansen-van Vuuren et al. 2022). In Ethiopia, religion and spirituality are inherent to cultural identity and influence views on child rearing, disability, health and well-being (Berie et al. 2020; Kahissay, Fenta & Boon 2018). As such, support providers of various expertises must understand and respect families’ values and beliefs and can tap into these as a resource for encouraging acceptance and commitment to children with disabilities, building resilience, and strengthening family and community relationships (Pandya 2017). Several studies from high-income contexts have identified the need to promote the inclusion of children with disabilities in religious congregations (Ault, Collins & Carter 2013; Carter et al. 2016) as well as consider spirituality and religion in special education (Ault 2010; Zhang 2012). However, further research is needed in low-income contexts, such as Ethiopia, to determine culturally acceptable approaches that support such community inclusion.

Some authors argue that spiritual beliefs can be detrimental to FQOL, promoting stigma when the cause of disability is attributed to spiritual factors (e.g. God’s curse, demonic activity) (Iyassu & McKinnon 2021; Teklemariam 2010). Although such beliefs certainly negatively influence FQOL, participants in our study argued that these beliefs derive from cultural traditions and superstitions, and rather than being condoned by religious institutions and spiritual leaders, are perhaps ‘warped interpretations of religion’ (Bertelli et al. 2019:2008). This disparity of beliefs highlights the need for widespread and dedicated education and awareness-raising, incorporating respected religious and community leaders as fellow disability advocates (Kahissay et al. 2018; Mihretu 2019; Teklemariam 2010). Health and rehabilitation workers may also provide a necessary link between traditional and biomedical approaches in Ethiopia through their understanding and relationships with local communities as well as health knowledge and training (Kahissay et al. 2018).

### Supporting the whole family to enhance family quality of life

This study demonstrated the inherently gendered responsibility of caring for children with disabilities as providers predominantly worked with mothers. Most FQOL studies focus on mothers’ perspectives (Mora et al. 2020) and in African contexts, mothers’ caregiving role is perhaps even more pronounced than in high-income contexts. However, disability affects the whole family and holistic, family-centred support promotes FQOL (Balcells-Balcells et al. 2019; Vanderkerken et al. 2019; Zuna et al. 2014). While support providers in contexts such as Ethiopia may need to be particularly attuned to mothers’ needs, they should also consider how to support and engage other family members such as fathers. Several African studies have explored why fathers are often less engaged in their children’s lives despite the benefits for children’s development and health; this was largely because of cultural identities where fathers are seen primarily as breadwinners and disability or weakness clashed with rigid perceptions of masculinity (Jackson & Andipatin 2021; Karisa, McKenzie & De Villiers 2021; Mavungu 2013). One South African study focused specifically on fathers’ experiences of caring for children with autism, finding both joys and challenges, but a need for greater support; fathers also expressed interest in peer-support (Pottas & Pedro 2016).

Participants in our study mentioned the importance of sibling relationships, which can influence FQOL. Yet each family situation is unique, and disability can have both positive and negative effects on sibling relationships (Correia & Seabra-Santos 2021; Kyrkou 2018; Paul et al. 2021). Research on siblings in African contexts is rare, but a South African pilot study showed primarily positive relationships between siblings with and without cerebral palsy (Mophosho, Widdows & Gomez 2009), while another South African study reported mainly negative experiences for siblings of children with severe disabilities (Opperman & Alant 2003). However, both studies affirmed the need for more family-focused support. Several reviews show inconsistent findings for interventions to support siblings of children with disabilities, largely because of the variability in siblings and therefore, their needs, as well as lack of methodological rigour in the included studies (Hartling et al. 2014; Tudor & Lerner 2014). Establishing sibling support groups and encouraging parents to openly communicate with age-appropriate information and prioritise quality time for siblings as well as the whole family together, could be beneficial for FQOL (Paul et al. 2021; Tsao, Davenport & Schmiege 2011). Other family members such as grandparents, can also provide support and enhance FQOL (Lee & Gardner 2010; Yang, Artman-Meeker & Roberts 2018), but they require support themselves, particularly in low-income contexts where grandparents (especially grandmothers) are often responsible for raising their grandchildren (Dolbin-MacNab & Yancura 2018). More research is needed in Ethiopia specifically to understand how to support various family members in culturally appropriate and feasible ways.

Establishing self-help and peer-support groups can support mothers emotionally, informationally and materially, empowering them to greater self-sufficiency (Aldersey, Turnbull & Turnbull 2016; Van Der Mark et al. 2019). Further research is needed, however, to explore how support groups could emotionally and practically benefit fathers, siblings, grandparents and other family members to build relationships and self-sufficiency and promote FQOL. Furthermore, the findings of this study confirm other African studies (including Ethiopia) showing the critical need for childcare to support mothers (Aldersey et al. 2020; Jansen-van Vuuren et al. 2021; McKenzie et al. 2020). Yet, childcare could also support the family more broadly by providing respite and allowing parents to work or spend quality time with other children, family or friends. Supporting strong family relationships through education and counselling (Masulani-Mwale et al. 2016; Paget et al. 2016) is another role for providers to build family resilience and enhance FQOL. As our participants suggested, engaging respected community and religious leaders and elders could help strengthen family relationships, provided they themselves receive appropriate training and support.

### Support providers need support too

This study highlights the inspiring resourcefulness, tenacity and compassion of Ethiopian support providers. However, they can only do so much with limited time and resources and all participants expressed a need for more support and collaboration to meet families’ needs. Collaborative efforts to address stigma and negative attitudes towards children with disabilities and their families are an overarching priority (Jansen-van Vuuren & Aldersey 2020; Jones, Seager & Yadete 2021; Tekola et al. 2020). Although the UNCRPD ratification was a positive step for disability rights in Ethiopia, ineffective policy implementation and enforcement hinder the full realisation of FQOL; Iyassu and McKinnon (2021) call for greater government accountability and action. Reducing disability stigma would not only benefit families but also support providers, by improving resource allocation, incentives and respect. Support providers from every sector need additional training (including opportunities and funding) to improve awareness, attitudes and skills as they work amongst people with disabilities, their families and communities (Jansen-van Vuuren & Aldersey 2018; Smith, Papadakis & Munnik 2022; Tilahun et al. 2019). Several participants highlighted the urban–rural divide where rural families face greater barriers from stigma and limited support. Considering the higher proportion of disability and poverty in rural areas, more resources and support are needed in rural areas to increase access to disability and health services, as well as education and employment opportunities for children and adults with disabilities (Geda et al. 2016; Hailemariam et al. 2019; Tekola et al. 2016).

### Limitations

Despite efforts to incorporate diverse perspectives of Ethiopian support providers, this study is not without limitations. Firstly, recruiting participants with the CBR programme’s assistance may have created bias and not fully portray the breadth of support providers involved with families of children with disabilities. However, this partnership provided important connections to participants who would otherwise have been difficult to contact. Many of the participants provided support in a more formal capacity; hence, interviewing other informal support providers (e.g. religious leaders, neighbours) could offer different and important insights. Future research could also compare informal and formal support for families, including roles, effectiveness and access to different types of support. Secondly, because of COVID-19 interviews were conducted using Zoom, with the potential for diminishing gestures and expressions and rapport. Despite many participants interviewing at the CBR centre, poor internet networks were a challenge, disrupting interviews, and sometimes making understanding difficult. Thirdly, the first author was not Ethiopian and could not speak Amharic; therefore, she did not have a deep understanding of the local context and required an interpreter for some of the interviews. Translation allowed a real-time conversation between the interviewer and participants so they could express themselves in their own language. However, interpreters influence how information is conveyed and meaning is potentially lost in translation (Chiumento et al. 2017; Resch & Enzenhofer 2018). Considering the pros and cons of professional versus ‘informal’ interpreters, the authors attempted to take advantage of both; their interpreter had no formal interpreting qualification, but had an excellent grasp of English and Amharic, communicated professionally and was familiar with the CBR programme.

## Conclusion

Disability support providers from diverse backgrounds described various approaches to supporting Ethiopian families of children with disabilities, affirming the centrality of spirituality, relationships (with family and community) and self-sufficiency (primarily through education and employment) for FQOL in this context. They were committed to families, recognising the enormous challenges and imperative need for support; however, they also expressed frustration and disappointment at the limited resources and negative societal attitudes towards disability. An overarching role and priority to enhance FQOL was raising disability-awareness through educating families and communities. Participants urged collaborative engagement and action because ‘the issue [of disability] is for all human beings’. This requires commitment from governments, Non-government organisations (NGOs), OPDs, disability advocates, support providers and families, to build an inclusive and accessible environment for all, where children with disabilities and their families can ultimately flourish.
